# The Effects of Cannabis Use Frequency and Episodic Specificity Training on the Recall of Specific and Rewarding Events

**DOI:** 10.3389/fpsyt.2021.643819

**Published:** 2021-07-07

**Authors:** Michael J. Sofis, Shea M. Lemley, Alan J. Budney

**Affiliations:** Center for Technology and Behavioral Health, Geisel School of Medicine, Dartmouth College, Lebanon, NH, United States

**Keywords:** cannabis (marijuana), episodic memory (EM), episodic specificity induction, reward, cannabis use disorder

## Abstract

**Background:** Growing evidence implicates subjective episodic memory, the retrieval of detailed, integrated, and personally relevant past events, as a marker of cognitive vulnerability in mental disorders. Frequent and problematic cannabis use is associated with deficits in objective episodic memory (verbal memory), but the relationship between subjective episodic memory deficits and frequency of cannabis use is unknown. Further, whether a brief intervention designed to enhance the specificity of event retrieval, such as the Episodic Specificity Induction (ESI), might effectively target such deficits among regular cannabis users is unexamined. This study was designed to examine subjective episodic memory as a potential marker of cognitive vulnerability among frequent cannabis users.

**Methods:** Active cannabis users (*n* = 133) recruited from Amazon Mechanical Turk or Qualtrics Panels were randomized to receive an ESI-control or ESI session and were separated into those who used cannabis 1–25 days in the past month (low to moderate frequency group) and those who used 26–30 days (high frequency group), which facilitated a low to moderate use/ESI-control group (*n* = 78), low to moderate use/ESI group (*n* =15), high-use/ESI-control group (*n* = 20), and high-use/ESI group (*n* = 20). Following the ESI or ESI-control intervention, participants selected four, positive events from the prior day, described the who, what, and where of the events, and rated how specific (vividness) and rewarding (enjoyable, importance, and exciting) each event was on a 0–100 scale. Four two-way ANCOVAs (demographics and problematic cannabis use covariates) were performed to examine the effects of frequency of cannabis use group and ESI group on the specificity and reward ratings.

**Results:** Lower vividness and excitement ratings were reported for those with high relative to low to moderate cannabis use frequency patterns (*p* < 0.05). Those who received ESI reported greater vividness, excitement, and importance ratings than the ESI-control group (*p* < 0.01). No significant interactions between frequency and ESI were found.

**Conclusion:** Findings from the current exploratory study provide initial evidence suggesting that more frequent cannabis use may be associated with the retrieval of less specific and rewarding events relative to less frequent users. Further, ESI may improve such deficits. Future studies that recruit larger and more clinically serious samples of cannabis users appear warranted.

## Introduction

Episodic memory, defined as the capacity to retrieve details of personal past events (1), is often sub-categorized as either a form of verbal memory (e.g., accurately recalling when you last spoke to a friend; objective episodic memory); or as the retrieval of detailed, integrated, and personally relevant past events [e.g., recalling the sensory, affective, and contextual details of when you last spoke to a friend; subjective episodic memory; (2)]. Deficits in subjective episodic memory are associated with a tendency to overlook specific contextual details in favor of overgeneralizing experiences into a single theme or central meaning (3). This failure to retrieve detailed characteristics of past events may inhibit the ability to vividly re-experience positive past events, simulate positive future events, and is associated with more frequent rumination, avoidance behaviors, and cognitive biases (4–6). Such deficits have been shown to predict the trajectory and the response to treatment in those with Major Depressive Disorder, Post-Traumatic Stress Disorder, and Schizophrenia (7–9). Moreover, interventions that prompt the practice of recalling detailed past events have been shown to improve the specificity of event retrieval and may mediate the effects of specificity training on reductions in depression symptoms (6). These findings suggest that specificity of event retrieval may function as a marker of cognitive vulnerability for mental disorders and that interventions which effectively target this construct may help improve treatment for clinical disorders associated with episodic memory deficits.

Frequent cannabis use and Cannabis Use Disorder (CUD) have been associated with risk for developing moderate to large deficits in objective episodic memory (10, 11). Laboratory studies have demonstrated a dose-dependent relationship between acute cannabis administration and performance on objective episodic memory tasks, and longitudinal studies have shown that within-person increases in frequency or severity of cannabis use problems correspond with greater decrements in objective episodic memory (11–14). Few, if any studies, however, have explored the relationship between subjective measures of episodic memory and cannabis use. It may be important to address such a gap in the literature because deficits in the ability to retrieve salient past events among those who use cannabis frequently corresponds with problems evaluating past events as rewarding. Further, less specific and rewarding retrieval of past events relates to greater devaluation of future rewards, known as delay discounting (DD), which is a risk factor associated with more frequent and problematic cannabis use and which has been shown to predict treatment outcomes for CUD (15–17). Reduced specificity when recalling past events also negatively impacts the ability to simulate detailed and rewarding future events (18), which may contribute to deficits in problem-solving and planning commonly observed in those who engage in frequent or problematic cannabis use. However, there is a need to test whether frequent cannabis use is associated with deficits in subjective episodic memory. Moreover, to better understand whether subjective episodic memory deficits may function as a treatment target for interventions that seek to reduce cannabis use, there is a need to examine whether subjective episodic memory can be enhanced in those who regularly use cannabis.

Episodic Specificity Induction (ESI) is a brief intervention that has been effectively used to enhance the specificity of event retrieval (1, 19). Episodic Specificity Induction prompts recollection of episodic details derived from a brief video through guided questions that increase the specificity of mentally constructed events (19). Episodic Specificity Induction has been shown to enhance the amount of detail individuals can recall from past events, which in turn may contribute to increases in positive affect and decreases in negative affect in healthy individuals (20). Episodic Specificity Induction has also shown to increase the number of alternative future events constructed during a simulation task (21). To date, no studies have explored how ESI affects subjective episodic memory in those who regularly use cannabis.

To test whether more frequent cannabis use is associated with deficits in subjective episodic memory, we compared event retrieval responses (i.e., reward and specificity ratings) between those with low to moderate relative to high frequency cannabis use patterns following an ESI intervention or an ESI control condition. We hypothesized that (1) those who used cannabis more frequently would retrieve less specific and rewarding events relative to those with less frequent use, and (2) that ESI would enhance specificity and reward ratings relative to ESI control. Lastly, we examined whether there was a significant interaction between ESI condition and frequency of use groups (low to moderate vs. high) such that ESI would enhance subjective episodic memory measures to a greater extent in high frequency relative to low to moderate frequency cannabis users. If observed, this would provide support that ESI may be a particularly helpful intervention for targeting subjective episodic memory deficits in among more frequent cannabis users. The goal of this study was to provide an initial validation for the role of subjective episodic memory as a potential treatment target for novel interventions designed to reduce cannabis use.

## Methods

### Procedures

The Institutional Review Board from Dartmouth College approved all procedures. All study sessions were administered remotely using Qualtrics survey software and participants were recruited using the crowdsourcing platform Amazon Mechanical Turk (mTurk) or Qualtrics Panel participants. All data described in the current study were derived from a single intervention session conducted in one of two studies.

Participants (*n* = 133) completed an ESI-control (*ESI-c)* session (*n* = 98; Study 1) or an *ESI* session (*n* = 35; Study 2). All intervention components that were administered across both studies were the same. mTurk participants who completed either study earned up to $7.50. Qualtrics Research Panel participants were compensated via standard procedures for the panels (exact amount varied and was unknown to us), however, Qualtrics representatives indicated that the compensation was similar in magnitude to that of our mTurk participants.

### Participants

Participants were recruited from Amazon Mechanical Turk (mTurk), an online crowdsourcing marketplace, in Study 1, and from both mTurk and Qualtrics Research Panels in Study 2. To be eligible for mTurk recruitment, workers participants had to have a 95% or higher approval rating on all previously submitted mTurk HITs and to have completed at least 100 Human Intelligence Tasks (HITs). Inclusion criteria for both sources included: reside in the U.S, age 18 or older, used cannabis in the last month (Study 1) or at least 10 days in the past month (Study 2), and used cannabis at least 100 lifetime days. To be inclusive of older cannabis users who increasingly use cannabis, participants older than 65 were included in the study to facilitate recruitment of a larger sample size. However, given previous evidence of age-specific differences in episodic memory (1), we controlled for age in the primary models conducted for this study. Within each of the two studies, participants were excluded if they missed two or more of three total attention checks or showed inconsistent responding.

#### Episodic Specificity Induction

Episodic Specificity Induction prompted recollection of episodic details through open-ended text responses related to the who (e.g., “Who was in the video?” “What were they wearing?”), what (e.g., “What was the video about?”), and when (“In what order did the events of the video occur?”) of the video (1, 22). The ESI involves watching a 2-min video of a woman giving a tour of her tiny house, and participants could not move to the next step in the training until the entire 2-min had elapsed (22). Participants then type answers to each of seven questions about episodic details from the tiny house video (e.g., “What did the people in the video look like?,” “What happened in the video, in order?”).

#### Episodic Specificity Induction-Control

Those in the ESI-c component watched the same tiny house tour video as those in the ESI condition. Unlike the ESI condition, those in the ESI-c condition answered seven questions designed to prompt semantic (external) details related to the video to control for the attention of participants and so that only the type of retrieval (episodic vs. semantic) was manipulated (e.g., “What did you think about the setting of the video?,” “How do you think it [the video] was made?”).

#### Event Retrieval

To assess the retrieval of events, participants were prompted using Episodic Recent Thinking [ERT, (23, 24)]. Episodic Recent Thinking prompts episodic thinking of positive events from yesterday during 3-h intervals [(25), 4–7 p.m., 1–4 p.m., 10–1 p.m., 7–10 a.m. (23, 24)]. Participants typed short answers to six questions about the episodic details of each event (e.g., “What were you doing?,” “Who were you with?,” “What were you tasting and smelling?”) to facilitate engaging in the rewarding and specific details of the events.

### Measures

#### Demographics and Substance Use

Table 1 shows distributions and descriptive statistics for demographic variables and cannabis use measures. Participants ranged from age 19–75 (*M* = 36.4 years, *SD* = 10.8), and 49% were female. Most participants were college educated (52%) and were employed full-time (67%). All cannabis use measures regarding use during the 30 days before the ESI/ESI-c session were ordinal in nature. Median number of days of cannabis use was 10–19 days per month (IQR = 1–2, 26–29 days).

**Table 1:** Demographic and participant characteristics.

**Demographic variables**	***Overall***	***ESI-c (n = 98)***	***ESI (n = 35)***	***p***
**Age** ***(M, SD)***	36.4 (11)	34.4 (1.1)	42.7 (2.3)	<0.01^a^
**Gender** ***(n, %)***				
Female	127 (48%)	48 (49)	21 (60)	0.30
**Level of education** ***(n, %)***				
College degree	135 (52)	52 (53)	14 (40)	0.33
**Employment (*****n*****, %)**				
Full-time		71 (72)	18 (51)	<0.01^a^
**Cannabis use variables**				
CUD *(n, %)*	140 (54)	56 (57)	18 (51)	0.68
Readiness to change *(M, SD)*	2.3 (2.5)	2.1 (2.5)	2.5 (1.7)	0.57
Days of use *(Mdn)*	10–19	6–9	26–29	<0.01^a^

“*Mdn” represents the exact median, “M” represents the mean, and “SD” represents the standard deviation. “CUD” indicates the proportion of participants who meet the cutoff score for screening positive for Cannabis Use Disorder (CUD) using the Cannabis Use Disorder Identification Test Short-Form (CUDIT-SF). “Readiness to Change” represents participants' readiness to reduce cannabis use which is based on a measure adapted from the readiness ruler (26). “Days of Use” represents the number of days of cannabis use in the past month. Instances of superscribed “a” denote statistically significant differences (p < 0.05) between ESI-c and ESI groups for the variable in question*.

Approximately half of the total sample (52%) met criteria for a cutoff score on the Cannabis Use Disorder Identification Task-Short Form (CUDIT-SF), which has been shown to be predictive of a CUD diagnosis (27). Participants answered the question, “How important is it for you to reduce your cannabis use?” using an 11-point visual analog scale (VAS) with anchors of “not” important at 0 and “very” important at 10. This assessment was adapted from the readiness ruler and was assessed pre and post-intervention (26). Participants averaged 2.3 on readiness to change cannabis use prior to the ESI/ESI-c intervention (*SD* = 2.5).

#### Specificity and Reward Ratings

For every event generated during ESI or ESI-c sessions, participants rated the excitement, enjoyment, and importance (i.e., how rewarding) and the vividness (i.e., specificity) on separate 100-point VASs. These ratings were considered a measure of engagement for each event (23). To create an average engagement score, all four engagement ratings were averaged for each participant.

### Analysis Plan

To examine potential differences in subjective episodic memory measures based on frequency of cannabis use responses on frequency of cannabis use days in the past month (i.e., 1–2, 3–5, 6–9, 10–19, 20–25, 26–29, all 30 days) were dichotomized into low to moderate frequency use (1–25 days; Low to Moderate Frequency Group) and more frequent use (26–30 days; High Frequency Group). These specific cutoff points for frequency of cannabis use were chosen in part because the median response ranged between 26 and 30 days of use in prior studies (17, 28). Further, because the modal response in the current study was 26–29 days of use, which was selected twice as frequently as the 20–25 days option, and the 10–19 days option was the least frequently selected option, the cut-off point of 26 days appeared to be the most data-based cutoff for this sample.

Demographic variables and cannabis-related variables were compared between ESI and ESI-c groups using chi-squared tests (categorical variables) and one-way ANOVAs (continuous variables). Those in the ESI condition were significantly older and were less likely to be employed full-time. No significant differences were observed between the two conditions on gender, education, the sum of the CUDIT-SF score, or readiness to change cannabis use. Age, employment, CUDIT-SF summed score, and gender were included as covariates in the main models of this study because age and employment differed between the two conditions and because both gender and problematic cannabis use have been consistently associated with deficits in episodic memory in past studies.

Four separate two-way ANCOVAs (ESI condition x frequency of use group) controlling for age, employment, CUDIT-SF summed score, and gender were performed to test for differences in specificity (vividness) and reward ratings (enjoyment, excitement, and importance) during ERT. ANCOVAs were used instead of a single MANOVA because each of the four dependent measures were highly correlated (*r*s = 0.54–0.77), which suggests the need to test for differences in separate models to avoid potential type I error.

## Results

**Figure 1:**
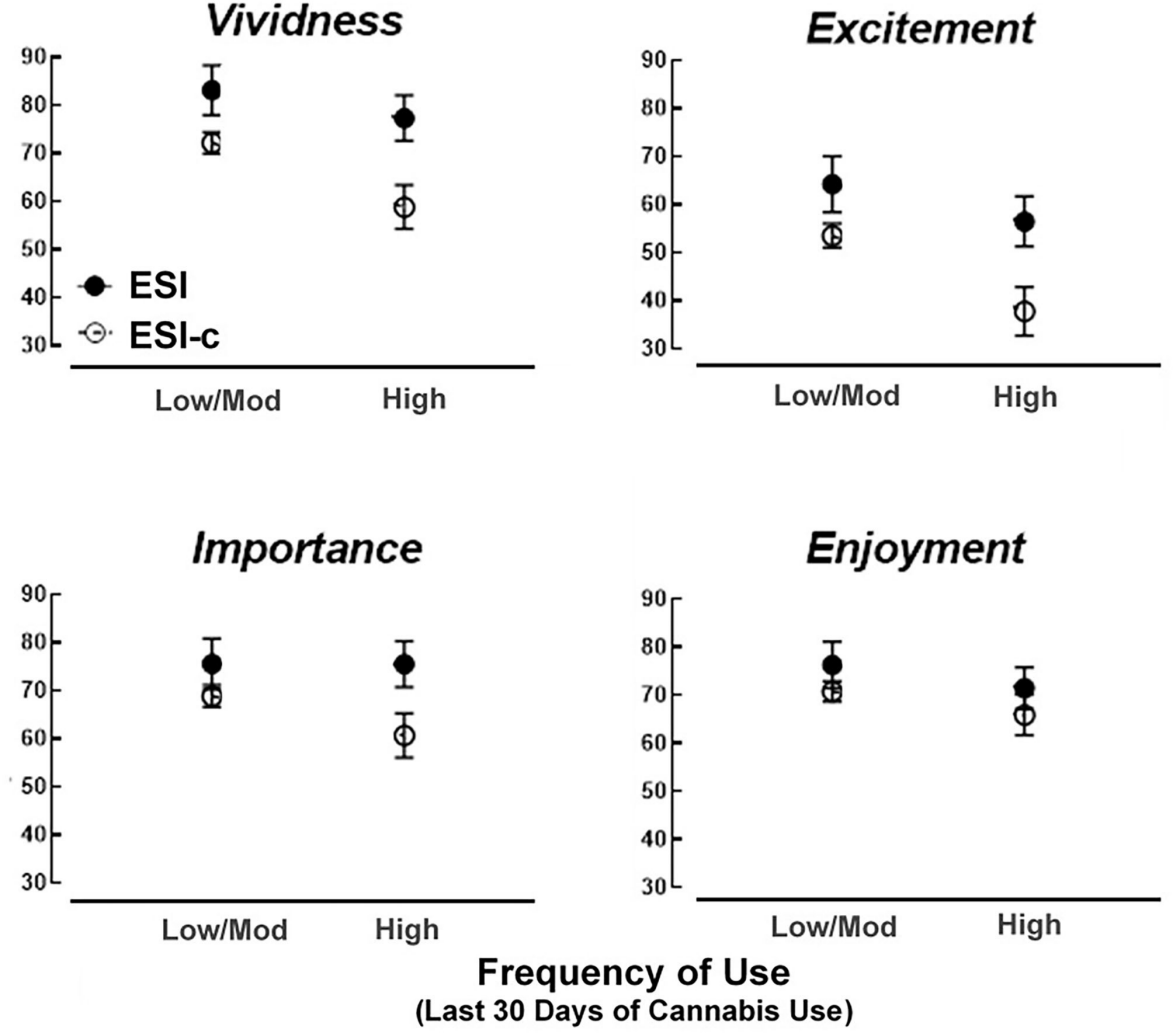
Subjective episodic memory ratings as a function of frequency of cannabis use and ESI/ESI-c groups.

Figure 1 details the results in adjusted means from two-way ANCOVAs for each event rating. For vividness ratings (specificity), there was a significant main effect of condition found such that those in the ESI condition demonstrated greater vividness ratings than those in the ESI-c condition [*F*_(1, 125)_ = 11.35, *p* < 0.01, *d* = 0.60]. There was a significant main effect of frequency found such that those in the high frequency group showed lower vividness ratings relative to the low to moderate frequency group [*F*_(1, 125)_ = 4.57, *p* < 0.05, *d* = 0.38]. A significant interaction effect for condition was not observed [*F*_(1, 125)_ = 0.80, *p* = 0.37, *d* = 0.16]. These findings suggest that ESI was associated with enhanced vividness ratings relative to ESI-c, that those in the high frequency cannabis use group showed lower vividness ratings than the low to moderate frequency group, but that ESI was not significantly more effective at enhancing vividness in high relative to low to moderate frequency cannabis use groups.

For the excitement ratings (reward), results of the two-way ANCOVA revealed greater ratings for those in the ESI than in the ESI-c condition [*F*_(1, 125)_ = 8.99, *p* < 0.01, *d* = 0.54] and lower ratings for those in the high frequency relative to low to moderate frequency group [*F*_(1, 125)_ = 5.45, *p* < 0.05, *d* = 0.42]. There was not a significant interaction between ESI and frequency of cannabis conditions [*F*_(1, 125)_ = 0.72, *p* = 0.40, *d* = 0.16]. These findings suggest that ESI was associated with enhanced excitement ratings relative to ESI-c, that those in the high frequency cannabis use group showed lower excitement ratings than those in the low to moderate frequency group, and that ESI was not significantly more effective at enhancing excitement in high relative to low to moderate frequency cannabis use groups.

For the importance ratings (reward), results of the two-way ANCOVA revealed greater ratings for those in the ESI condition than in the ESI-c condition [*F*_(1, 125)_ = 5.76, *p* < 0.05, *d* = 0.43], but there was not a significant difference in importance between those in the high frequency and low to moderate frequency groups [*F*_(1, 125)_ = 0.80, *p* = 0.37, *d* = 0.16] or a significant interaction [*F*_(1, 125)_ = 0.89, *p* = 0.35, *d* = 0.17]. These findings suggest that ESI was associated with enhanced importance ratings relative to ESI-c, that frequency of cannabis use was not associated with importance ratings, and that ESI was not significantly more effective at enhancing importance in the high relative to the low to moderate frequency cannabis use groups.

For the enjoyment ratings (reward), results of the two-way ANCOVA revealed that there were not any significant differences between ESI and ESI-c conditions [*F*_(1, 125)_ = 1.87, *p* = 0.17, *d* = 0.25], between those in the high frequency and low to moderate frequency groups [*F*_(1, 125)_ = 1.35, *p* = 0.25, *d* = 0.21] or a significant interaction [*F*_(1, 125)_ = 0.0001, *p* = 0.99, *d* = 0.00]. Due to the lack of a significant main effect or interaction effect, no follow-up ANCOVA models were performed. These findings suggest that enjoyment ratings were not associated with frequency of cannabis use, were not enhanced by ESI, and were not differentially augmented in those who reported high relative to low to moderate frequency cannabis use.

## Discussion

This study examined whether more frequent cannabis use is associated with deficits in subjective episodic memory (i.e., specificity and reward retrieval ratings), and whether an ESI intervention could enhance subjective episodic memory relative to an ESI control condition. We observed lower ratings in high frequency users for vividness and excitement relative to the low to moderate frequency users, which would be suggestive of deficits associated with more frequent cannabis use. Those receiving the ESI intervention showed greater vividness, excitement, and importance ratings relative to those who received the ESI-c intervention, which suggests that the ESI may improve subjective measures of episodic memory among cannabis users. Importantly, no significant interaction effects between ESI condition and frequency of cannabis use groups were found, which suggests that the subjective episodic memory measures were not enhanced by ESI to a greater extent in high frequency relative to low to moderate frequency cannabis users in this study. One interpretation of the current findings is that specificity (vividness) of event recall may be impacted to a greater extent by frequent cannabis use than by how rewarding recalled events are perceived to be, thus providing a potentially more relevant treatment target for interventions designed to reduce cannabis use. Such an interpretation would be congruent with evidence suggesting that episodic memory deficits are more pronounced than reward-related deficits among regular cannabis users (10).

The failure to observe a consistent effect of cannabis use frequency and ESI condition across all four reward ratings was unexpected given that more specific event recall often corresponds with more positive perceptions of past and potential future events (20). One potential reason for this finding is the current study prompted recall of events from the prior day, in contrast to other studies which have provided a broader time frame for participants to choose (29). Thus, there may have been less variability in the reward ratings in this study because there was a more restricted opportunity to recall past positive events. Although reward and specificity of event recall have been significantly related to each other in prior studies, there is also some evidence suggesting that the two constructs are bidirectionally related (4). Thus, it may be necessary to also target valuation of retrieved events to enhance cognitive processes such as DD and emotional regulation by prompting the elaboration of positive and rewarding features of the video events presented by the ESI.

This study is the first to our knowledge to demonstrate the initial efficacy of a brief intervention to improve engagement in episodic retrieval in frequent cannabis users. The current digital version of ESI was completed in 10 min on average, which suggests the feasibility of administering the ESI in real-world environments. Our findings are congruent with other brief intervention studies that observed improvement in specificity of events among those with Major Depressive Disorder and Schizophrenia (6, 30). Of potential importance to the health behavior treatment field, is to determine if ESI can improve engagement in Episodic Future Thinking (EFT), a brief intervention currently being tested for reducing nicotine, alcohol, and cannabis use and improving healthy food choices (22–24, 31). Episodic Future Thinking is an intervention that prompts participants to create and imagine positive, personally relevant future events and is thought to be a product of episodic memory processes and the ability to focus on the future (18, 32). Through strengthening episodic memory processes, administering ESI prior to EFT may improve engagement in EFT, thus improving DD and potentially increasing the impact on reductions in cannabis use and other substances (22).

Several limitations of this study warrant note. Data from the ESI-c and ESI were derived from two separate studies and participants were recruited through two separate online mechanisms (i.e., Mechanical Turk and Qualtrics Panels), which may have influenced the findings due to variations in the sample characteristics between the two recruitment sources. The impact of this sampling strategy was minimized as the studies used the same experimental conditions and similar inclusion criteria and all variables that were significantly different between the two conditions were controlled for in analyses. The modest sample size posed another limitation. Statistical power was not optimal and raises concern about the reliability and generality of the findings, particularly in relation to the lack of significant interactions between ESI condition and frequency groups observed. Future studies that recruit larger samples and randomize participants to roughly equivalent group sizes appear warranted. Approximately half of the participants in this sample met criteria cutoff scores for CUD using a validated screening tool; however, they were not formally diagnosed with CUD, and so the relevance of CUD in the context of the current study should be further explored in a clinical CUD sample. Also, participants in the current study reported relatively high frequency cannabis use patterns and the clinical validity of the specific cutoff point used (i.e., <26 days, 26–30 days) has not been empirically established. Even in the low to moderate frequency cannabis group, participants could have used 5 days a week, which may limit the generality of these findings to individuals who use cannabis less frequently.

Despite these limitations, our findings suggest that subjective episodic memory deficits should continue to be explored as a potentially important marker of cognitive vulnerability. If such deficits are indeed ubiquitous with frequent and problematic cannabis use, they may provide new treatment targets for CUD interventions and potentially help inform the development of novel treatment approaches.

## Data Availability Statement

The raw data supporting the conclusions of this article will be made available by the authors, without undue reservation.

## Ethics Statement

The studies involving human participants were reviewed and approved by Dartmouth Committee for the Protection of Human Subjects. The patients/participants provided their written informed consent to participate in this study.

## Author Contributions

MS and AB performed study conceptualization and design. MS performed data collection and analysis. SL performed programming of study methods and analysis. MS and AB performed writing and SL performed editing. All authors contributed to the article and approved the submitted version.

## Conflict of Interest

The authors declare that the research was conducted in the absence of any commercial or financial relationships that could be construed as a potential conflict of interest.
